# Molecular Signature of Aluminum Hydroxide Adjuvant in Ovine PBMCs by Integrated mRNA and microRNA Transcriptome Sequencing

**DOI:** 10.3389/fimmu.2018.02406

**Published:** 2018-10-23

**Authors:** Endika Varela-Martínez, Naiara Abendaño, Javier Asín, Maialen Sistiaga-Poveda, Marta Maria Pérez, Ramsés Reina, Damián de Andrés, Lluís Luján, Begoña M. Jugo

**Affiliations:** ^1^Department of Genetics, Physical Anthropology and Animal Physiology, Faculty of Science and Technology, University of the Basque Country (UPV/EHU), Bilbao, Spain; ^2^Department of Animal Pathology, Veterinary Faculty, University of Zaragoza, Zaragoza, Spain; ^3^Institute of Agrobiotechnology (CSIC-UPNA-Government of Navarra), Navarra, Spain

**Keywords:** adjuvant, aluminum hydroxide, vaccination, transcriptome, miRNA, RNA-seq, sheep, PBMCs

## Abstract

There have been few *in vivo* studies on the effect of aluminum hydroxide adjuvant and its influence on the immune response to vaccination. In this study, lambs received a parallel subcutaneous treatment with either commercial vaccines containing aluminum hydroxide or an equivalent dose of this compound only with the aim of identifying the activated molecular signature. Blood samples were taken from each animal at the beginning and at the end of the experiment and PBMCs isolated. Total RNA and miRNA libraries were prepared and sequenced. After alignment to the Oar3.1 reference genome and differential expression with 3 programs, gene enrichment modeling was performed. For miRNAs, miRBase and RNAcentral databases were used for detection and characterization. Three expression comparisons were made: vaccinated animals at the beginning and at the end of the treatment, adjuvanted animals at the same times, and animals of both treatments at the end of the experiment. After exposure to both treatments, a total of 2,473; 2,980 and 429 differentially expressed genes were identified in vaccinated animals, adjuvanted animals and animals at the end of both treatments, respectively. In both adjuvant and vaccine treated animals the *NF-*κ*B* signaling pathway was enriched. On the other hand, it can be observed a downregulation of cytokines and cytokine receptors in the adjuvanted group compared to the vaccinated group at the final time, suggesting a milder induction of the immune response when the adjuvant is alone. As for the miRNA analysis, 95 miRNAs were detected: 64 previously annotated in *Ovis aries*, 11 annotated in *Bos taurus* and 20 newly described. Interestingly, 6 miRNAs were differentially expressed in adjuvant treated animals, and 3 and 1 in the other two comparisons. Lastly, an integrated miRNA-mRNA expression profile was developed, in which a miRNA-mediated regulation of genes related to DNA damage stimulus was observed. In brief, it seems that aluminum-containing adjuvants are not simple delivery vehicles for antigens, but also induce endogenous danger signals that can stimulate the immune system. Whether this contributes to long-lasting immune activation or to the overstimulation of the immune system remains to be elucidated.

## Introduction

Aluminum compounds have been used as adjuvants for nearly 90 years in veterinary and human vaccines. Aluminum hydroxide, aluminum phosphate and aluminum sulfate constitute the main forms of aluminum used as adjuvants. Despite its widespread use, the mechanism of how aluminum-based adjuvants exert their beneficial effects is still not fully understood. Moreover, they occasionally can cause adverse reactions (1). Gherardi et al. (2) described an inflammatory muscle disorder in humans characterized by a distinctive pattern of intramuscular inflammation that demonstrated the presence of aggregates of aluminum-containing macrophages and was linked to inoculation with aluminum-containing vaccines (3); today this disease is known as macrophagic myofasciitis (MMF). Several studies with animal models have also concluded that aluminum hydroxide-containing vaccines can lead to local tissue damage and neurobehavioral changes similar to MMF (4). In sheep, a form of the autoimmune/autoinflammatory syndrome induced by adjuvants (ASIA) (5) has been described as linked to repetitive inoculation with aluminum-containing vaccines. This syndrome was extensively observed after compulsory vaccination against the bluetongue virus of ruminants in 2008 (6).

Until recently, DNA microarrays were the primary tools used in molecular toxicology for the evaluation of drugs, but recently microarrays have also been used to analyze vaccines (7). The basis of this method lies in the quality, immunogenicity and reactogenicity of vaccines in expression profiling data. This method can be highly informative, fast and very sensitive. Unlike methods based on the hybridization of microarrays, RNA sequencing (RNA-seq) uses ultrasequencing technologies to determine transcriptomic profiles, that is, to detect and accurately quantify RNA molecules that originate in a genome at a certain time point. The sequencing of the transcriptomes using next-generation RNA-seq is an optimal tool for a precise and holistic analysis of the loci expressed in cells and tissues. Recent studies show that this technique is superior to other methods of transcriptome analysis due to its large dynamic range and its low technical variability (8). In addition, RNA-seq is not restricted to the known annotation of the genome, but allows the identification of functionally relevant unknown loci, which is very useful in genomes with imperfect annotations, as is the case for many species of domestic animals (9).

Few studies have used new RNA sequencing technologies to monitor the immune response to vaccination. Yang et al. (10) analyzed the liver transcriptome using RNA-seq to clarify the mechanisms of the host regarding the protector effect of a possible vaccine and its immunogenicity. Demasius et al. (11) monitored the immune response to vaccination using an inactivated vaccine for neonatal bovine pancytopenia. In both cases, new genes or routes involved in the immune response to vaccination were detected and demonstrated the importance of understanding this response in the development of new vaccines and their components.

miRNAs are increasingly being identified as key players in the immune system, regulating processes, such as the development, differentiation and function of immune cells. Several miRNAs have been identified in the different immune cell types, regulating a number of responses. An interesting pattern has also emerged where a single miRNA, such as miR-155, may influence global immune responses through its effect on macrophages, dendritic cells, and B and T lymphocytes through the direct regulation of distinct target genes (12).

Until now, there have been many *in vitro* but few *in vivo* studies on the influence of aluminum hydroxide adjuvant and its influence on the immune response to vaccination. Understanding how cells interact with adjuvants *in vivo* is crucial to characterize the mechanisms of action of this adjuvant and will be critical in the rational design of effective diagnostic tools and vaccines against many diseases (13).

Thus, the main objective of this study was to identify the molecular signature activated by vaccines and adjuvants in the form of aluminum hydroxide in sheep, providing insight into the mechanisms underlying the immune response, by combining the molecular information provided by RNA sequencing of both mRNA and miRNA in an *in vivo* experiment.

## Materials and methods

### Animals

All experimental procedures were approved and licensed by the Ethical Committee of the University of Zaragoza (ref: PI15/14). Requirements of the Spanish Policy for Animal Protection (RED53/2013) and the European Union Directive 2010/63 on the protection of experimental animals were always fulfilled. Rasa Aragonesa pure breed lambs were selected from a single pedigree flock of certified good health at 3 months old and did not undergo any vaccination before the experiment. The flock analyzed in this study was established at the experimental farm of the University of Zaragoza and was always maintained indoors, with ideal controlled conditions of housing, management and diet. The animals were kept 2 months to acclimatize to the new environment so they were 5 months old when the experiment started. For the purpose of the present work, they were randomly distributed in different treatment groups, *n* = 7 each. Each group received a parallel subcutaneous treatment with either commercial vaccines containing aluminum hydroxide [Al (OH)_3_] as adjuvant (Group Vac) or aluminum hydroxide only (Group Adj; Alhydrogel, CZ Veterinaria, Spain), always inoculating with the equivalent dose of aluminum applied in the vaccinated group. Nine different vaccines were used, and a total of 19 inoculations were applied in each group throughout 16 different inoculation dates, thus entailing a total amount of 81.29 mg of aluminum per animal. Intervals between inoculations ranged from 17 to 100 days (mean = 31.3 ± 22.1 days). The complete study lasted 475 days, from February 2015 to June 2016. Table S1 includes details of the commercial vaccines used and the inoculation protocol that was applied.

For RNA-seq analysis, a total of 6 animals were included (3 sheep inoculated with vaccines and 3 sheep inoculated with aluminum) and for validation of the sequencing data, 8 animals were included (4 sheep treated with vaccines and 4 sheep treated with aluminum; Table 1).

**Table 1:** Samples used in RNA-seq and RT-qPCR study.

**Treatment**	**Animals (6)**	**Time**	**Samples**
**RNA-seq**			
Vaccine	121, 124, 125	T0	121-A, 124-A, 125-A
		Tf	121-B, 124-B, 125-B, 125-B^*^
Aluminum	111, 114, 116	T0	111-A, 114-A, 116-A
		Tf	111-B, 114-B, 116-B
**Treatment**	**Animals (8)**	**Time**	**Samples**
**RT-qPCR**			
Vaccine	122, 123, 126, 127	T0	122-A, 123-A, 126-A, 127-A
		Tf	122-B, 123-B, 126-B, 127-B
Aluminum	112, 113, 115, 117	T0	112-A, 113-A, 115-A, 117-A
		Tf	112-B, 113-B, 115-B, 117-B

* *Same RNA sample obtained with a conventional trizol extraction method*.

### Blood collection and RNA extraction

For the isolation of ovine peripheral blood mononuclear cells (PBMCs), blood was collected from the jugular vein of 14 Rasa Aragonesa sheep. Blood samples were taken from each animal at the beginning (day 0, T0), before any vaccination, and at the end of the treatment (day 475, Tf), which was 5 days after the last inoculation. Blood was collected into heparinized Vacutainer tubes (Becton, Dickinson and Company, Sparks, MD), transferred into 50-ml centrifuge tubes and diluted 1:2 in HBSS. Twenty-five milliliters of blood:HBSS were layered over 10 ml of Ficoll-Paque (1.084 g/cm^3^) (GE HealthCare Bio-Sciences, Uppsala, Sweden) in 50-ml centrifuge tubes. The cells were centrifuged at 900 × g for 30 min to separate erythrocytes and polymorphonuclear cells from PBMCs. PBMCs were collected from the HBSS-Ficoll-Paque interface, washed with HBSS by centrifugation at 400 × g for 10 min, lysed in 1 ml of Trizol and stored at −80°C until further use.

Total RNA was extracted from PBMCs using an RNA Clean & Concentrator™-5 kit (Zymo Research, Irvine, CA, USA) following manufacturer's instructions and stored at −80°C. RNA quantity and purity were assessed with a NanoDrop 1000 Spectrophotometer (Thermo Scientific Inc., Bremen, Germany). The RNA integrity and concentration were assessed with a 2100 Bioanalizer (Agilent Technologies, Santa Clara, CA, USA). Two numeric parameters concerning RNA integrity were estimated, the 28S/18S (ribosomal RNA) ratio and the RNA integrity number (RIN value). The RNA samples with a RIN value >7.5 and a 260/280 ratio >1.8 were used.

### RNA sequencing

Total RNA-seq libraries were prepared according to the TruSeq Stranded Total RNA kit with Ribo-Zero Globin (Illumina, San Diego, CA, USA) to deplete the samples of cytoplasmic and mitochondrial rRNA and globin mRNA. The miRNA-seq libraries were prepared according to the TruSeq Small RNA library prep kit (Illumina). Total RNA and miRNA libraries were sequenced on a HiSeq2000 sequencer and HiSeq2500 sequencer, respectively. RNA-seq was conducted for a total of 13 samples, with a mean sequencing depth of 70 million 76 base pair (bp) paired-end reads at CNAG (Centro Nacional de Análisis Genómico, Barcelona, Spain). miRNA-seq included 12 samples, with a mean sequencing depth of 17 million 50 bp single-end reads at CRG (Centro de Regulación Genómica, Barcelona, Spain).

### Total RNA expression analysis

First, a quality check was performed on the raw data files with FASTQC [v0.11.5] (14) to assess the most appropriate read quality filtering and trimming. The following criteria were used with Trimmomatic [v0.36] (15): (1) remove adaptor sequences with the “palindrome” mode for paired-end data, allowing up to two mismatches; (2) remove reads in which the average Phred quality score within a sliding window of five nucleotides falls below 20; and (3) remove reads with a length <36 nucleotides. The data were checked again with FASTQC to ensure that the filtering was adequate.

The STAR RNA-seq aligner [v2.5.2b] (16) was used to align clean reads to the *Ovis aries* genome build Oar3.1 [version 89.31] (17) using the 2-pass mode. For each library, the featureCounts software from the SourceForge Subread package [v1.5.0-p1] (18) was applied to assign uniquely aligned fragments to annotated genes in a strand-specific manner. Once the expression levels were obtained they were evaluated by a set of plots from the NOISeq package [v2.20.0] (19) and by principal component analysis (PCA) to detect potential biases and contamination.

Differential gene expression analysis was performed using three different R packages within Bioconductor: edgeR [v3.18.1] (20, 21), DESeq2 [v1.16.1] (22), and limma [v3.32.2] (23). The DESeq2 package performs independent filtering, but for edgeR and limma a cutoff for filtering lowly expressed genes was set at 2 CPM (counts per million). Prior to the differential expression, a batch effect correction package, SVA [v3.24.0] (24), was applied to remove unwanted variation, and the surrogate variables were incorporated into the testing model.

RNA-seq counts were modeled by a generalized linear model, keeping in mind that not all samples were collected from independent subjects. From every subject, two blood samples were collected: at the start of the experiment (T0) and at the end of the experiment (Tf). The following variables were used in the model: *time* (T0 or Tf), *treatment* (complete vaccine [Vac] or adjuvant only [Adj]), *sample* (indicates the samples that come from the same individual), and *SVA covariates* (surrogate variables calculated by sva). The model included the *treatment* factor, the *batch* variable and the interactions *treatment* × *sample* (because there were different animals in each treatment), and *treatment* × *time* (to account for the treatment-specific time effects). Differential expression analyses were performed for the time points, considering the treatment group (Vac Tf vs. Vac T0 and Adj Tf vs. Adj T0), and for the treatments at the end of the experiment (Adj Tf vs. Vac Tf).

In DESeq2 and edgeR the read counts follow a negative binomial distribution, with a gene-specific dispersion parameter. In contrast, limma transforms the read-counts to log2(CPM) values and models the mean-variance relationship with precision weights (the “voom” approach). The differentially expressed genes (DEGs) were selected as those with an adjusted *p*-value (using the Benjamini-Hochberg method) threshold of <0.05 and a fold change value of >1.5 or <0.667. Only those genes that were identified as DEGs by all of the three programs were selected for further analysis.

To search for overrepresented gene functions in the lists of DEGs, gene enrichment analyses were conducted using the Gene Ontology (GO) database with PANTHER [v12.0] (25) and the Kyoto Encyclopedia of Genes and Genomes (KEGG) database with DAVID [v6.8] (26). Enriched terms were considered statistically significant with an adjusted *p*-value threshold of <0.05.

### miRNA expression analysis

For small RNA sequencing reads, all adaptor sequences were first removed, and then low-quality reads and reads shorter than 16 bp were filtered out. For the subsequent analyses, some of the sRNAtoolbox (27) modules were applied. The sRNAbech module was used to map the sequences to the *Ovis aries* reference genome Oar3.1, to profile the expression of small RNAs and to predict novel miRNAs. The program uses bowtie (28) behind the scenes to map all the sequences to the reference genome, and it searches in the miRBase [v21] (29) database for known miRNAs in sheep. Furthermore, Rfam data was used to identify other small RNAs originating from rRNA, tRNA, snRNA, and snoRNA and exclude them from the analysis. The remaining sequences were searched against the mature miRNAs of human and other species, including cow, goat and mouse, in miRBase to identify miRNA homologs. For the discovery of new miRNAs, the remaining sequences were used to predict their folding secondary structure and, if a hairpin structure was predicted, their free energy of hybridization. Ultimately, the predicted new miRNAs were searched in the RNACentral [v6] database with blastn to ascertain if they have been previously identified.

The differential miRNA expression analysis was performed using the edgeR package with the same model as for the total RNA-seq. The SVA package was first applied to remove unwanted variation. The differentially expressed miRNAs were selected as those with an adjusted *p*-value (by the Benjamini-Hochberg method) threshold of <0.05 and a fold change value of >1.5 or <0.667.

The mRNAconsTarget module was used to identify potential miRNA target genes with the miRanda (30) and PITA (31) algorithms. At the same time, the target prediction algorithm TargetScan (32) was applied independently, using a total of 3 distinct target prediction algorithms. To select trustworthy target genes, the following cutoffs were selected: in miRanda, a pairing score >150 and an energy score < −15; in PITA, an energy score < −15; and in TargetScan, a contex++ score < −0.7. All of the programs returned a vast list of targets. To reduce false positives and select candidate targets, only those genes that were common across the three programs were selected for further analysis.

Next, integrating the total RNA and miRNA analyses, only those target genes that were negatively correlated with the specific miRNA were selected. Correlations between miRNA and mRNA expression values were determined using the R statistical software [v3.4.1]. A test for association between paired samples using the Spearman's rank correlation coefficient was applied with the R *cor. test* function. The obtained *p*-values were adjusted due to multiple comparisons with the Benjamini-Hochberg method using a threshold of < 0.05 to indicate significant miRNA-mRNA pairs.

### Validation of differential total RNA and miRNA expression

To validate changes that were identified by the RNA-seq experiments, the relative expression levels of 9 genes (*CNTLN, EGR2, GPRC5C, HGF, NRXN2, SAMD4B, SKAP2, TREM1, WDR5B*) and 3 miRNAs (*oar-let-7b, oar-miR-19b, oar-miR-25*) that were selected based on significant changes seen in the RNA-seq and miRNA-seq analyses were verified by qPCR. For quantification of mRNA transcripts, primers were designed using the PrimerQuest and OligoAnalyzer tools of Integrated DNA Technologies (IDT). *GAPDH, ATPase, ACTB* and *TFRC* were used as reference genes. For quantification of miRNAs, primers were designed using the Qiagen platform. *U6* snRNA, *oar-miR-30d*, and *oar-miR-191* were used as internal standards. These last two miRNAs were selected for their expression stability in our samples. Table S2 shows the list of the amplified ovine genes and miRNAs and the corresponding primer sequences. The real-time qPCR amplification of cDNA pools was accomplished using PowerUp™ SYBR™ Green Master Mix (Applied Biosystems, Foster City, CA, USA) in a 10 μl final volume reaction, according to the manufacturer's instructions. qPCR reactions were conducted on a QuantStudio® 3 detection system (Applied Biosystems) under the following conditions: 1 cycle of 50°C for 2 min, 1 cycle of 95°C for 2 min, 40 cycles of denaturation at 95°C for 15 s, annealing at 60°C for 60 s, and a dissociation curve to measure the specificity of the amplification. Appropriate controls (no template and no retrotranscription) were included. Primer concentrations that did not produce nonspecific fragments or primer dimers and generated the lowest Ct values were selected for the final analysis.

The expression study was based on the analysis of mRNA and miRNA expression with Fludigm's BioMark HD Nanofluidic qPCR system technology combined with GE 48.48 Dynamic Arrays IFC. qPCR was performed on a BioMark HD system using Master Mix SsoFastTM EvaGreen® Supermix with Low ROX (Bio-Rad Laboratories, Hercules, CA, USA). The expression analysis using the Fluidigm Biomark HD Nanofluidic qPCR system was performed at the Gene Expression Unit of the Genomics Facility, in the General Research Services (SGIKER) of the UPV/EHU. Ct value and real-time PCR analysis was carried out with Fluidigm Real-Time PCR Analysis software [v3.1.3]. PCR efficiency calculation and correction, reference gene and miRNA stability analysis and normalization were accomplished with the GenEx software of MultiD [v5.4]. Most of the genes and miRNAs showed high amplification efficiencies, with mean values of 96 and 99%, respectively. The stability of candidate reference genes and miRNAs was analyzed using both the NormFinder (33) and GeNorm (34) algorithms integrated in GenEx. The two most stable genes were *ACTB* and *GAPDH*, and normalization was performed using these two reference genes. The two most stable miRNAs were *oar-miR-30d* and *oar-miR-191*, and normalization was performed using these two miRNAs.

Changes in gene and miRNA expression (*n*-fold) or relative quantification (RQ) were determined by the Δ(Δ*Ct*) method. Based on the sequencing results, three comparisons were made: Vac Tf vs. Vac T0, Adj Tf vs. Adj T0, and Adj Tf vs. Vac Tf. The results are expressed as relative quantifications and fold changes, which was standardized by log2 transformation. Normal distribution was checked using the Shapiro-Wilk test in the IBM SPSS statistical package [v24]. Changes in expression between different groups (Vac Tf vs. Vac T0, Adj Tf vs. Adj T0, and Adj Tf vs. Vac Tf) were compared with the Tukey HSD or Games-Howell *post-hoc* test (ANOVA) or with the nonparametric Kruskal-Wallis test of the SPSS package. In all analyses, the differences were considered significant when the *p*-value was <0.05.

## Results

### Summary statistics for RNA-seq data

A total of 13 RNA-seq libraries were sequenced, giving a mean value of 69.7 million raw paired-end reads per library. After filtering for adaptor sequences and low-quality fragments, a mean of 68.5 million reads (98.33%) remained for subsequent analyses. Alignment of the filtered reads to the *Ovis arie*s reference genome (Oar3.1) yielded a mean value of 52.7 million read pairs (76.95%) mapping to unique loci per library, 11.4 million read pairs (16.70%) mapping to multiple loci and 4.3 million read pairs (6.35%) not mapping to any loci in the genome. Only the uniquely mapped reads were used for subsequent analyses, and a mean value of 36.1 million read pairs (68.42%) were successfully assigned to annotated genes in a strand-specific manner.

### Analysis of differential gene expression from RNA-seq data

Gene coverage analysis revealed that of the 27,054 annotated *Ovis aries* genes in Ensembl (release 89), 21,274 (78.63%) were expressed with at least one sequence read count in at least one of the 13 RNA-seq libraries. Detected genes whose expression was lower than 2 CPM reads and could be found in <6 individual libraries were treated as lowly expressed genes and were filtered out from the differential expression analysis. These cutoffs were selected after checking that lower values introduced genes whose expression was minimal and were expressed in only a few animals of each group. Such lowly expressed genes do not provide enough statistical evidence in the differential expression analysis for a reliable judgment to be made (35). In total, 11,395 genes (42.12%) were used in the differential expression analysis.

Prior to any other analysis, the selected genes were used to generate a PCA to visualize the 13 samples and to check if there was any type of bias in the data. To accurately measure biological variability and to obtain the correct statistical inference in the analysis, the *svaseq* function from the SVA package was applied to account for batch variables and unknown factors while preserving the variation of interest. A new PCA was obtained with the corrected data (Figure S1A). In this PCA, the samples were grouped according to the treatment condition.

After the batch effect correction, all 11,395 genes that passed the filtering were used for differential expression analysis in three different programs (edgeR, DESeq2, and limma), designating the intersection between the results of the programs (with a *p*-value < 0.05 and a fold change >1.5 or < 0.667) as true DEGs. In the Vac Tf vs. Vac T0 comparison, 2,473 DEGs (Figure S2A) were identified, of which 1,208 and 1,265 displayed increased and decreased expression, respectively. Showing a similar pattern, in the Adj Tf vs. Adj T0 comparison, 2,980 DEGs (Figure S2B) were identified, of which 1,474 and 1,506 were upregulated and downregulated, respectively. Furthermore, in the Adj Tf vs. Vac Tf comparison, 429 DEGs (Figure S2C) were identified, of which 132 were upregulated and 297 were downregulated. A detailed list of the DEGs can be seen in Table S3.

**Figure 1:**
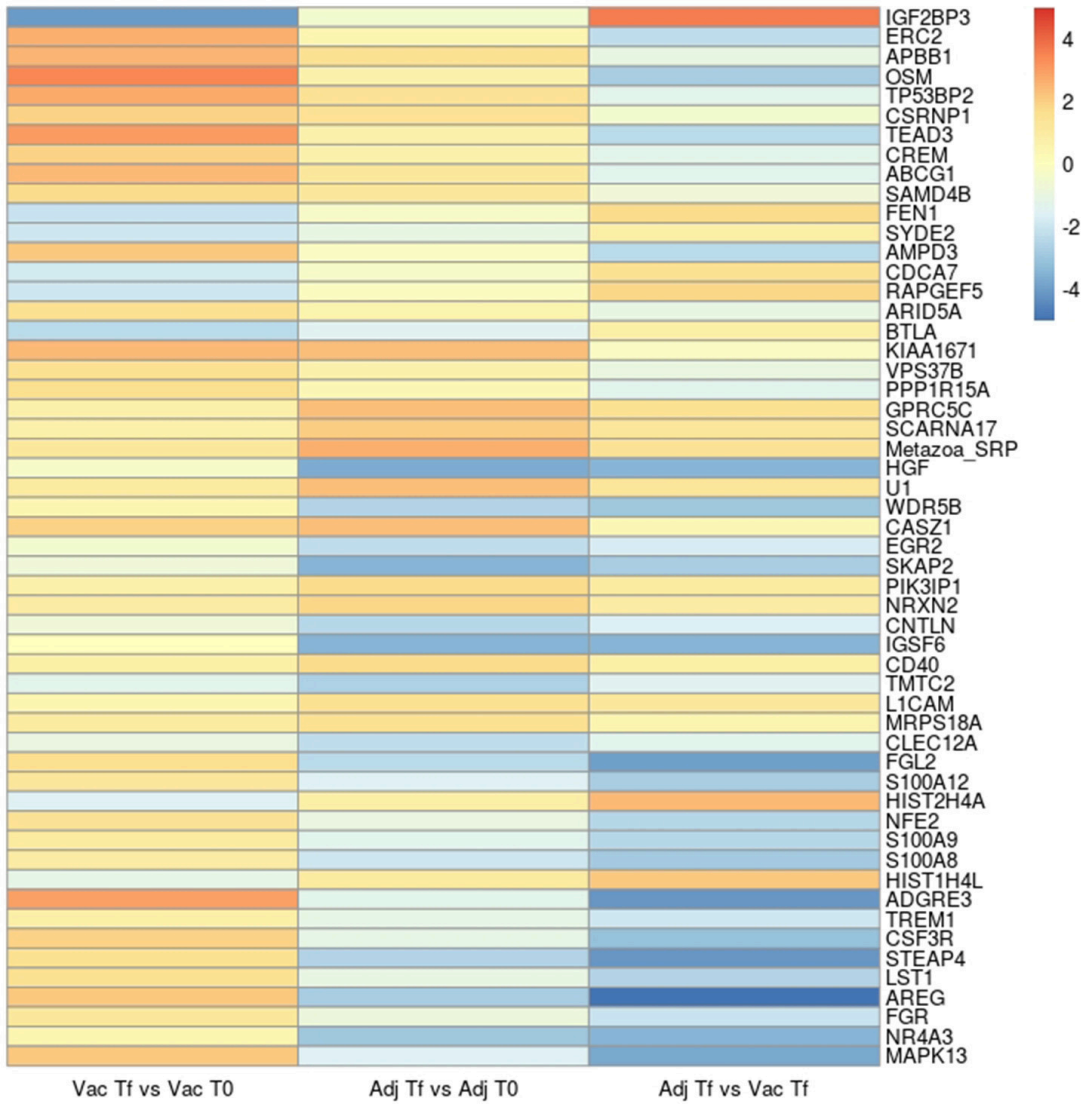
Heatmap with the log2(Fold change) of the top 10 significant up- and down-regulated genes in the Vac Tf vs. Vac T0, Adj Tf vs. Adj T0, and Adj Tf vs. Vac Tf comparisons. The genes were selected from those found differentially expressed in 3 different programs: limma, edger, and DESeq2.

The top 10 most significant up- and downregulated genes of each comparison are shown as a heat-map (Figure 1). Within the most up- or downregulated genes are factors that are clearly related to apoptosis (*TP53BP2, CSRNP1, TEAD3, CDCA7, PPP1R15A*), immune response (*OSM, AMPD3, BTLA, SKAP2, IGSF6, LST1, FGR, MAPK13*), regulation of inflammatory response (*CD40, S100A12, ADGRE3, TREM1, STEAP4, NR4A3*), DNA replication and repair (*FEN1, HIST1H4L*), cell growth (*ARID5A, VPS37B, HGF, CSF3R*), cell adhesion and cell-cell signaling (*NRXN2, CLEC12A, AREG*), nervous system development (*RAPGEF5, CASZ1, EGR2, L1CAM*), and a gene involved in the pathogenesis of Alzheimer's disease (*APBB1*).

To validate the RNA-seq data, 9 mRNAs (*CNTLN, EGR2, GPRC5C, HGF, NRXN2, SAMD4B, SKAP2, TREM1, WDR5B*) were verified using the Fluidigm Biomark HD Nanofluidic qPCR system. Fold changes in gene expression between the different groups calculated by RT-qPCR are shown in Table S4. Although the fold change values for the expression of some genes measured by RNA-seq or RT-qPCR were different, in terms of fold change direction, the gene expression patterns of most genes (6 in Vac Tf vs. Vac T0, 9 in Adj Tf vs. Adj T0, and 7 in Adj Tf vs. Vac Tf) (81.5%) were reproducible by the RT-qPCR analysis.

### Functional annotation and classification for RNA-seq data

**Figure 2:**
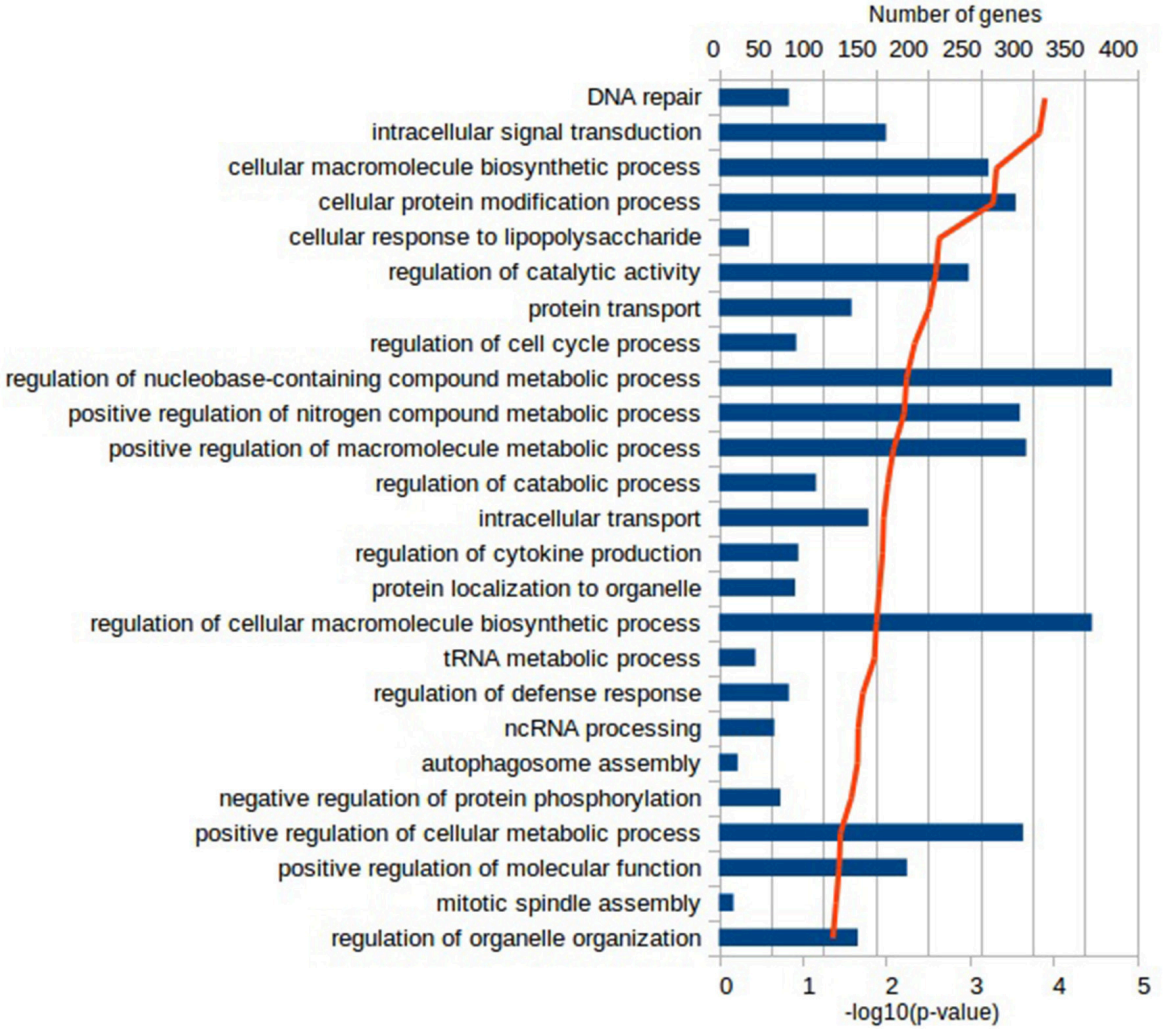
The most enriched GO terms in the Biological Process ontology in the Vac Tf vs. Vac T0 comparison in PANTHER with the Fisher's exact test and Benjamini-Hochberg False Discovery Rate correction. The blue bars depict the number of genes in the enriched terms, while the red line the adjusted-*p*-values.

**Figure 3:**
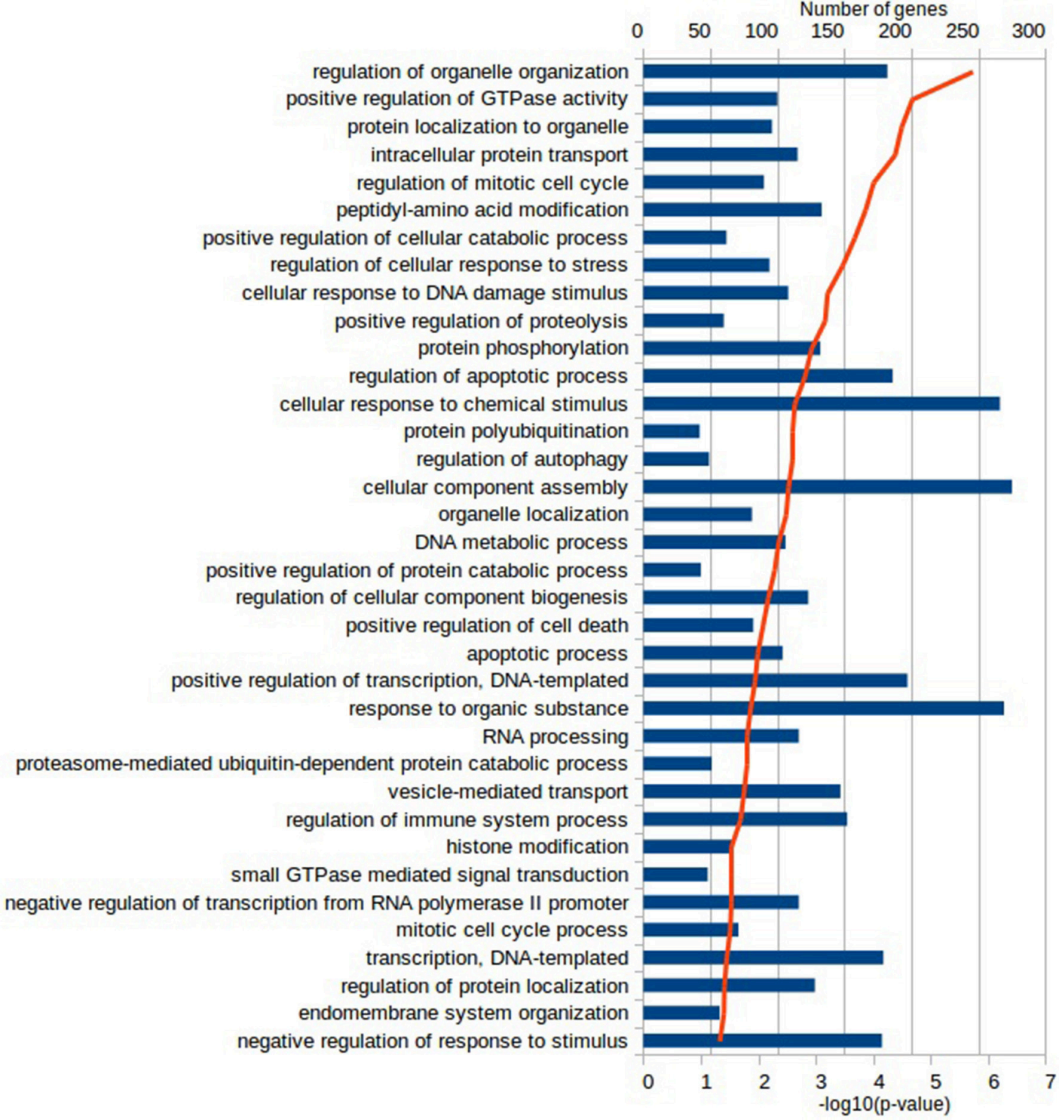
The most enriched GO terms in the Biological Process ontology in the Adj Tf vs. Adj T0 comparison in PANTHER with the Fisher's exact test and Benjamini-Hochberg False Discovery Rate correction. The blue bars depict the number of genes in the enriched terms, while the red line the adjusted-*p*-values.

**Figure 4:**
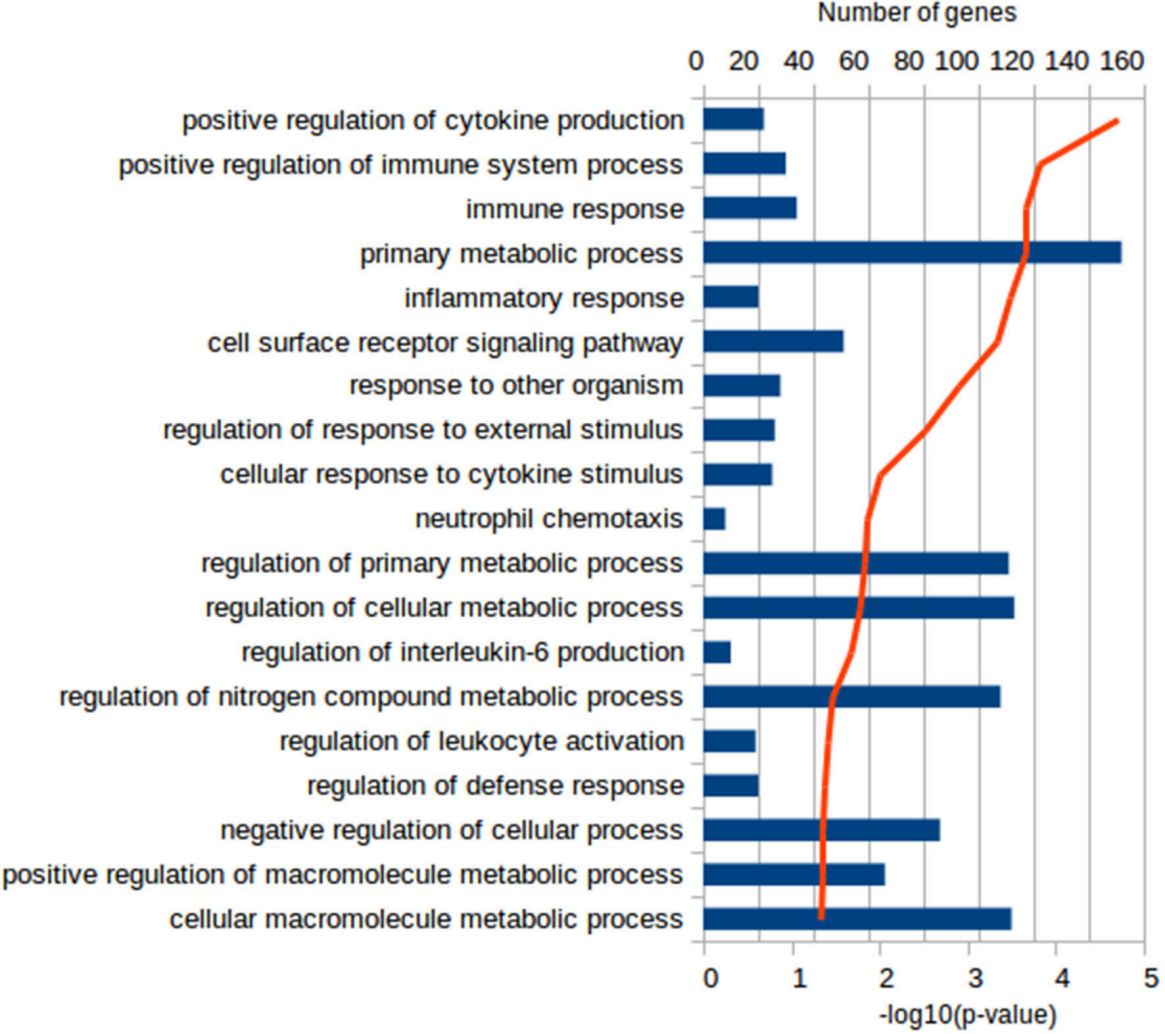
The most enriched GO terms in the Biological Process ontology in the Adj Tf vs. Vac Tf comparison in PANTHER with the Fisher's exact test and Benjamini-Hochberg False Discovery Rate correction. The blue bars depict the number of genes in the enriched terms, while the red line the adjusted-*p*-values.

Functional characterization of the DEGs was performed with PANTHER to identify enriched GO terms in the three domains: Cellular Component (CC), Molecular Function (MF), and Biological Process (BP). In the Vac Tf vs. Vac T0 comparison, 46 significantly overrepresented GO terms (with an adjusted *p*-value < 0.05) were identified in total. Among the top ranked Biological Processes were *intracellular signal transduction (GO:0035556), cellular response to lipopolysaccharide (GO:0071222), regulation of cytokine production (GO:0001817), DNA repair (GO:0006281)* and *regulation of defense response (GO:0031347)* (Figure 2). In addition, in the Adj Tf vs. Adj T0 comparison, there were 72 overrepresented GO terms, including *positive regulation of GTPase activity (GO:0043547), regulation of cellular response to stress (GO:0080135), cellular response to DNA damage stimulus (GO:0006974), positive regulation of proteolysis (GO:0045862), regulation of apoptotic process (GO:0042981), cellular response to chemical stimulus (GO:0070887), regulation of autophagy (GO:0010506)* and *regulation of immune system process (GO:0002682)* (Figure 3). Finally, in the Adj Tf vs. Vac Tf comparison, there were 23 overrepresented GO terms, including *positive regulation of cytokine production (GO:0001819), positive regulation of immune system process (GO:0002684), inflammatory response (GO:0006954), immune response (GO:0006955), regulation of response to external stimulus (GO:0032101), cellular response to cytokine stimulus (GO:0071345)*, and *neutrophil chemotaxis (GO:0030593)* (Figure 4).

**Figure 5:**
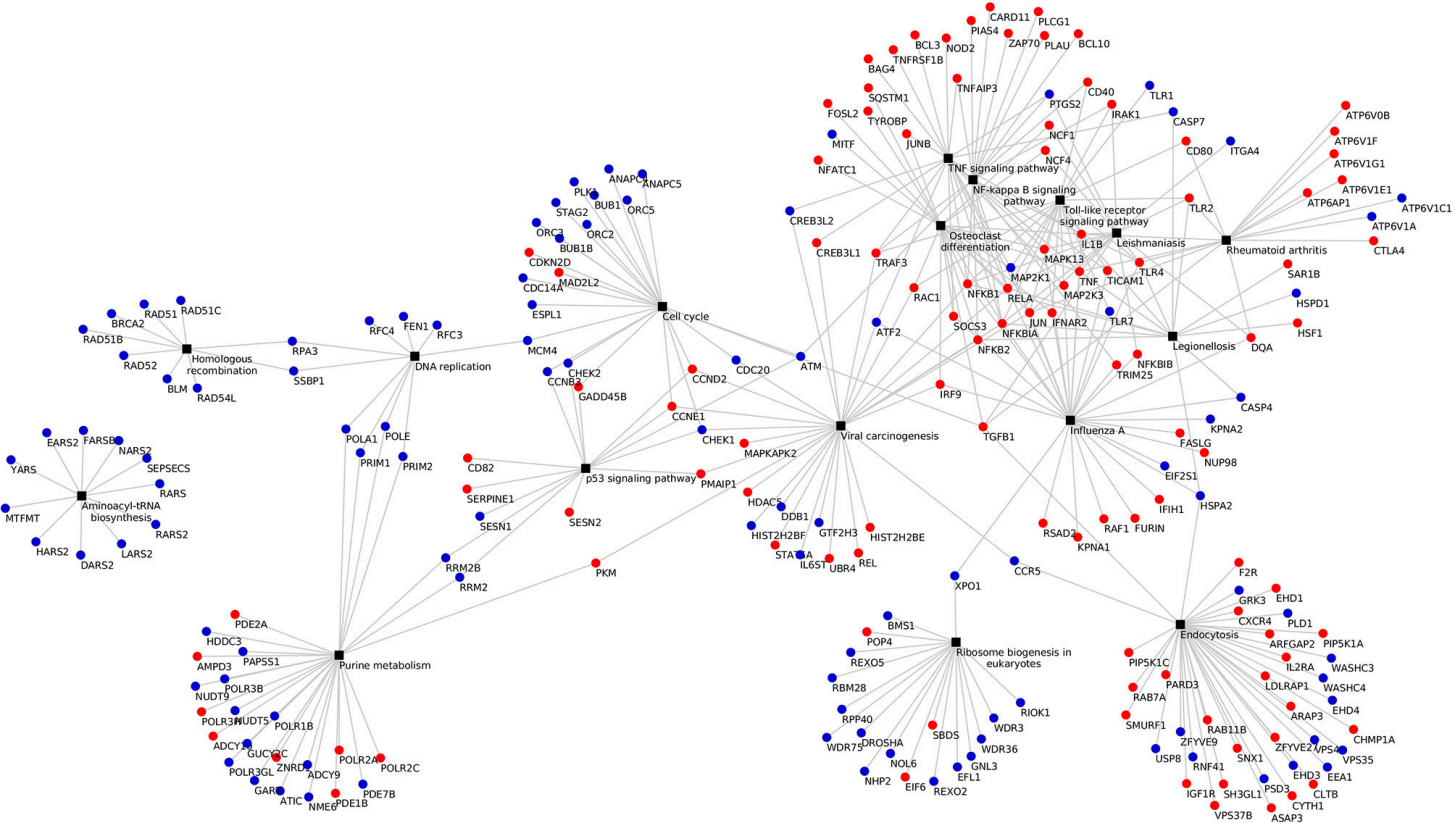
Enriched KEGG pathways in the Vac Tf vs. Vac T0 comparison in DAVID with EASE Score (a modified Fisher's exact test) and Benjamini-Hochberg False Discovery Rate correction. Black boxes represent different pathways and the points the differentially expressed genes in each pathway, up-regulated ones in red and down-regulated ones in blue.

**Figure 6:**
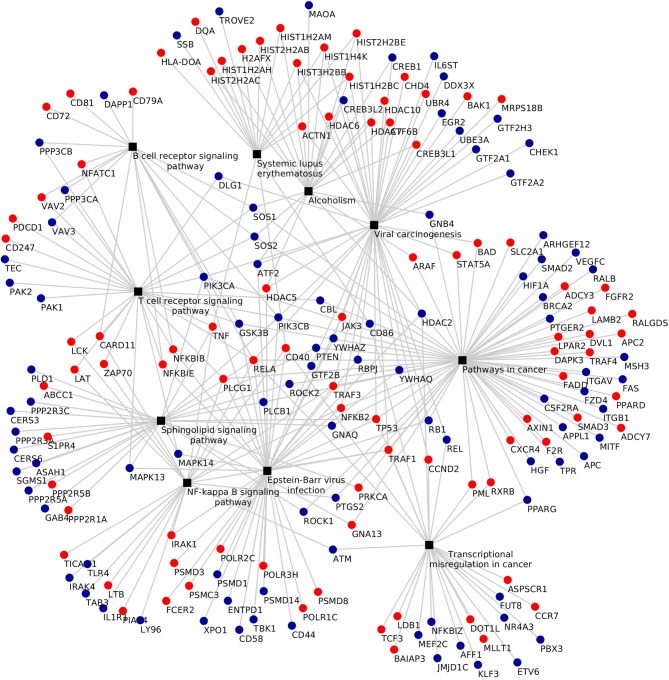
Enriched KEGG pathways in the Adj Tf vs. Adj T0 comparison in DAVID with EASE Score (a modified Fisher's exact test) and Benjamini-Hochberg False Discovery Rate correction. Black boxes represent different pathways and the points the differentially expressed genes in each pathway, up-regulated ones in red and down-regulated ones in blue.

**Figure 7:**
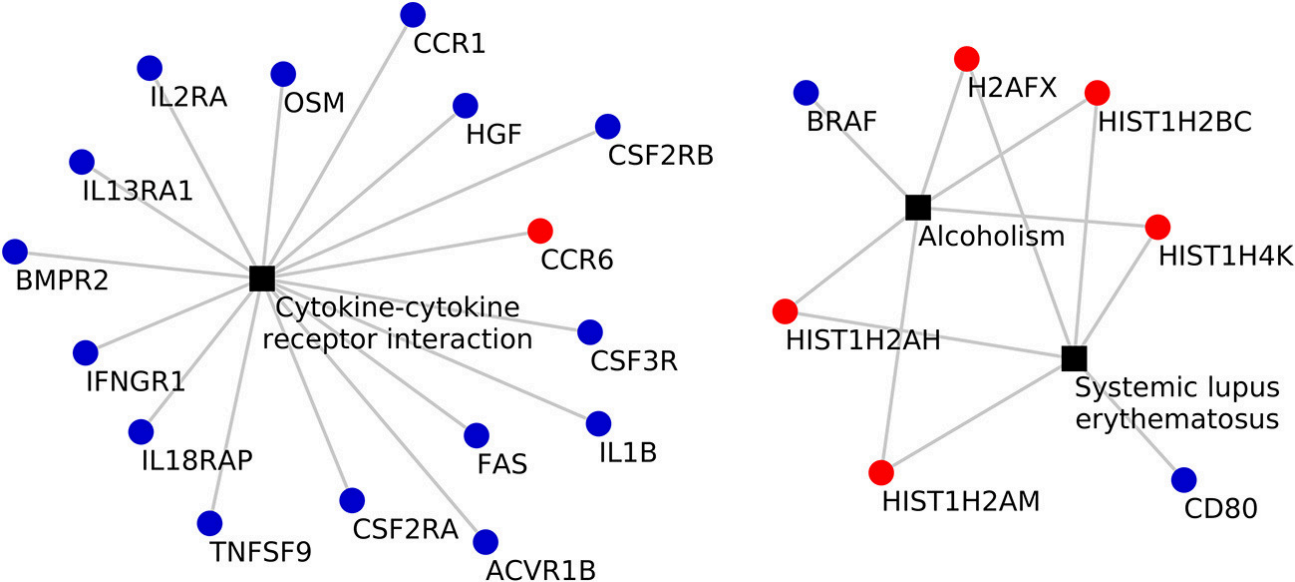
Enriched KEGG pathways in the Adj Tf vs. Vac Tf comparison in DAVID with EASE Score (a modified Fisher's exact test) and Benjamini-Hochberg False Discovery Rate correction. Black boxes represent different pathways and the points the differentially expressed genes in each pathway, up-regulated ones in red and down-regulated ones in blue.

KEGG pathway analysis of the DEGs using DAVID tools revealed an overrepresentation of genes with roles in the immune system, inflammatory response and autoimmune diseases. In both the adjuvant- and vaccine-treated animals, the *NF-*κ*B signaling pathway* was enriched. Other enriched pathways exclusive to each treatment were: *TNF signaling pathway, Toll-like receptor signaling pathway, p53 signaling pathway, DNA replication, purine metabolism* and *endocytosis* in Vac Tf vs. Vac T0 (Figure 5); *T cell receptor signaling pathway* and *B cell receptor signaling pathway* in Adj Tf vs. Adj T0 (Figure 6); and *cytokine-cytokine receptor interaction* in Adj Tf vs. Vac Tf (Figure 7). Notably, nearly all cytokines and cytokine receptors in the KEGG pathway are downregulated in the Adj Tf group compared to Vac Tf, except for CCR6.

**Figure 8:**
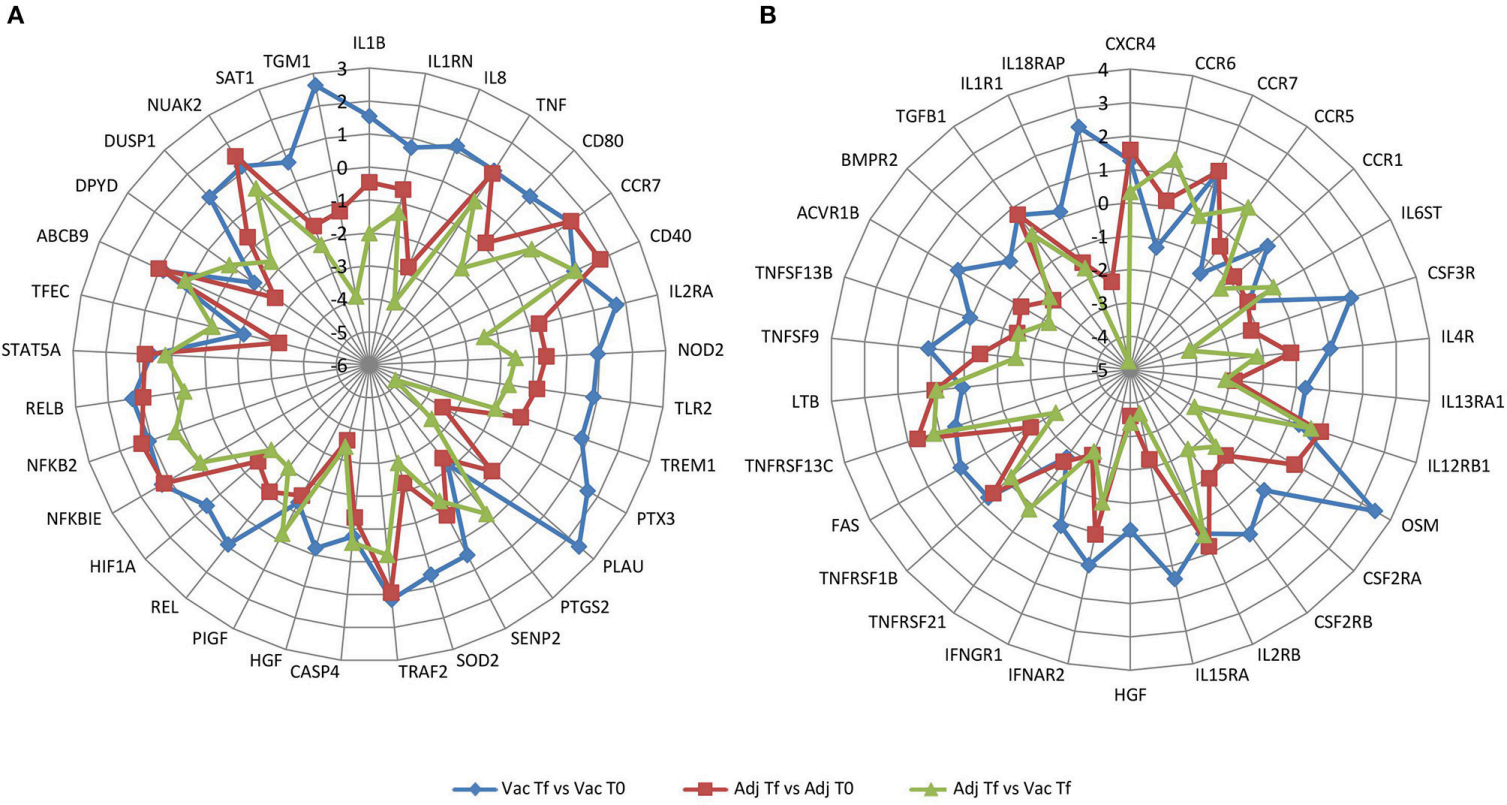
Radar plot with the log_2_(FoldChange) of overrepresented genes in **(A)** *NF-*κ*B signaling pathway* and in **(B)** *cytokine-cytokine receptor interaction pathway* in the Vac Tf vs. Vac T0 (blue), Adj Tf vs. Adj T0 (red), and Adj Tf vs. Vac Tf (green) comparisons.

Next, to summarize which genes were overrepresented in two of the most enriched pathways, *NF-*κ*B signaling pathway* in vaccine- and adjuvant-treated animals and *cytokine-cytokine receptor interaction* in the Adj Tf vs. Vac Tf comparison, a radar plot was produced (Figures 8A,B). Within the *NF-*κ*B* target genes are cytokines/chemokines (*IL1B, IL1RN, IL8*, and *TNF*), immunoreceptors (*CD80, CCR7, CD40, IL2RA, NOD2, TLR2, TREM1*), acute phase proteins (*PTX3, PLAU*), stress response genes (*PTGS2, SENP2, SOD2*), regulators of apoptosis (*TRAF2, CASP4*), growth factors (*HGF, PIGF*), transcription factors (*REL, HIF1A, NFKBIE, NFKB2, RELB, STAT5A, TFEC*), and enzymes (*ABCB9, DPYD, DUSP1, NUAK2, SAT1, TGM1*) (Figure 8A). Within the cytokine-cytokine receptor interaction pathway there are chemokines (*CXCR4, CCR6, CCR7, CCR5, CCR1, IL8*), haematopoietins (*IL6ST, CSF3R, IL4R, IL13RA1, IL12RB1, OSM*) and genes belonging to the *PDGF* family (*CSF2RA, CSF2RB, IL2RA, IL2RB, IL15RA, HGF*), interferon family (*IFNAR2, IFNGR1*), *TNF* family (*TNFRSF21, TNFRSF1B, FAS, CD40, TNFRSF13C, TNF, LTB, TNFSF9, TNFSF13B*), *TGFB* family (*ACVR1B, BMPR2, TGFB1*), and *IL1* family (*IL1R1, IL18RAP, IL1B*) (Figure 8B).

Vaccination induced a clear upregulation of *IL1B, IL2RA*, and *PTX3*, consistent with the induction of an ongoing immune response against the vaccine. In contrast, inoculation with aluminum alone generally downregulated the mRNA expression of several proinflammatory genes, including *IL1B, IL8, TLR2, NOD2*, or *IL2RA*, suggesting a milder induction of the immune response (Figure 8A). Cytokine receptor interaction evidenced the induction of *IL18RAP*, involved in sensing the proinflammatory *IL18* cytokine, and *CSF3R*, which is the receptor for granulocyte colony stimulation factor (*G-CSF*), a key cytokine that controls myeloid cell function.

### Summary statistics for miRNA-seq data

The 12 miRNA-seq libraries were sequenced, giving a mean value of 17.2 million raw paired-end reads per library. After filtering for adaptor sequences and low-quality fragments, a mean of 14.2 million reads (82.38%) remained for subsequent analyses. Alignment of the filtered reads to the *Ovis aries* reference genome (Oar3.1), allowing a maximum of 20 multiple mappings per read, yielded a mean value of 12.9 million read pairs (91.40% of the filtered reads) mapping to different loci per library.

### Analysis of differential gene expression from miRNA-seq data

After alignment, all sequences were searched in miRBase to identify annotated miRNAs and in Rfam to identify other small RNAs originating from rRNA, tRNA, snRNA and snoRNA for the purpose of excluding them from the analysis. In total, 56 annotated *Ovis aries* miRNAs were expressed with at least one sequence read count in at least one of the 12 sample RNA-seq libraries. Furthermore, new miRNAs were predicted with the unassigned reads, obtaining 39 new miRNAs (Table S5) with at least one sequence read in a sample. Of these 39 new miRNAs, 11 were similar to other miRNAs from *Bos Taurus* in miRBase. Moreover, the predicted new miRNAs were searched in RNACentral database with blastn, and another 8 miRNAs were linked to *Ovis aries* miRNAs found in other studies.

The length distribution of all miRNAs were checked, and the majority of the reads were 21–24 nucleotides in length, a range distribution common in mammalian miRNAs (36). Detected miRNAs whose expression was lower than 1 CPM and were found in <6 individual libraries were treated as lowly expressed miRNAs and were filtered out from the differential expression analysis. In total, 64 miRNAs were used in the differential expression analysis.

Prior to any other analysis, the selected miRNAs were used to generate a PCA to visualize the 12 samples and to check if there was any type of bias in the data. To account for batch variables and other unknown factors, similar to the total RNA-seq analysis, some surrogate variables were calculated with SVA. A new PCA was obtained with the corrected data (Figure S1B). In this PCA, the samples were grouped according to treatment condition.

After the batch effect correction, all 64 miRNAs that passed the filtering were used for differential expression analysis with edgeR. A total of 3 (*oar-miR-125b, oar-miR-99a*, and *new-miR-2284ab-5p*), 6 (*oar-miR-25, oar-miR-379-5p, oar-miR-411a-5p, oar-miR-16b, oar-miR-19b*, and *oar-let-7b*) and 1 (*new-miR-2284ab-5p*) differentially expressed miRNAs (with a *p*-value < 0.05 and a fold change >1.5 or < 0.667) were identified in the Vac Tf vs. Vac T0, Adj Tf vs. Adj T0 and Adj Tf vs. Vac Tf comparisons, respectively (Table 2).

**Table 2:** List of differentially expressed miRNAs detected by edgeR with a FDR-adjusted *p*-values of ≤0.05.

**Vaccine Tf vs. Vaccine T0**			**Adjuvant Tf vs. Adjuvant T0**			**Adjuvant Tf vs. Vaccine Tf**		
**miRNA**	**logFC**	**FDR**	**miRNA**	**logFC**	**FDR**	**miRNA**	**logFC**	**FDR**
new-miR-2284ab-5p	−8,613	1,063E-06	oar-miR-25	2,171	2,003E-03	new-miR-2284ab-5p	12,072	1,483E-03
oar-miR-125b	2,225	6,024E-04	oar-miR-379-5p	−4,208	2,003E-03	–	–	–
oar-miR-99a	1,654	1,369E-02	oar-miR-411a-5p	−6,556	6,028E-03	–	–	–
–	–	–	oar-miR-16b	1,732	2,023E-02	–	–	–
–	–	–	oar-miR-19b	−1,799	2,635E-02	–	–	–
–	–	–	oar-let-7b	1,576	3,259E-02	–	–	–

To validate the miRNA-seq data, 3 miRNAs (*oar-let-7b, oar-miR-19b, oar-miR-25*) were verified using the Fluidigm Biomark HD Nanofluidic qPCR system. Fold changes in miRNA expression as calculated by RT-qPCR are shown in Table S4. Validation results confirmed the upregulated expression of 2 miRNAs (*oar-let-7b* and *oar-miR-25*) and the downregulated expression of *oar-miR-19b*. The miRNA data from RNA-seq and RT-qPCR showed a high degree of concordance.

### miRNA target prediction and integration of miRNA and mRNA expression profiles

**Figure 9:**
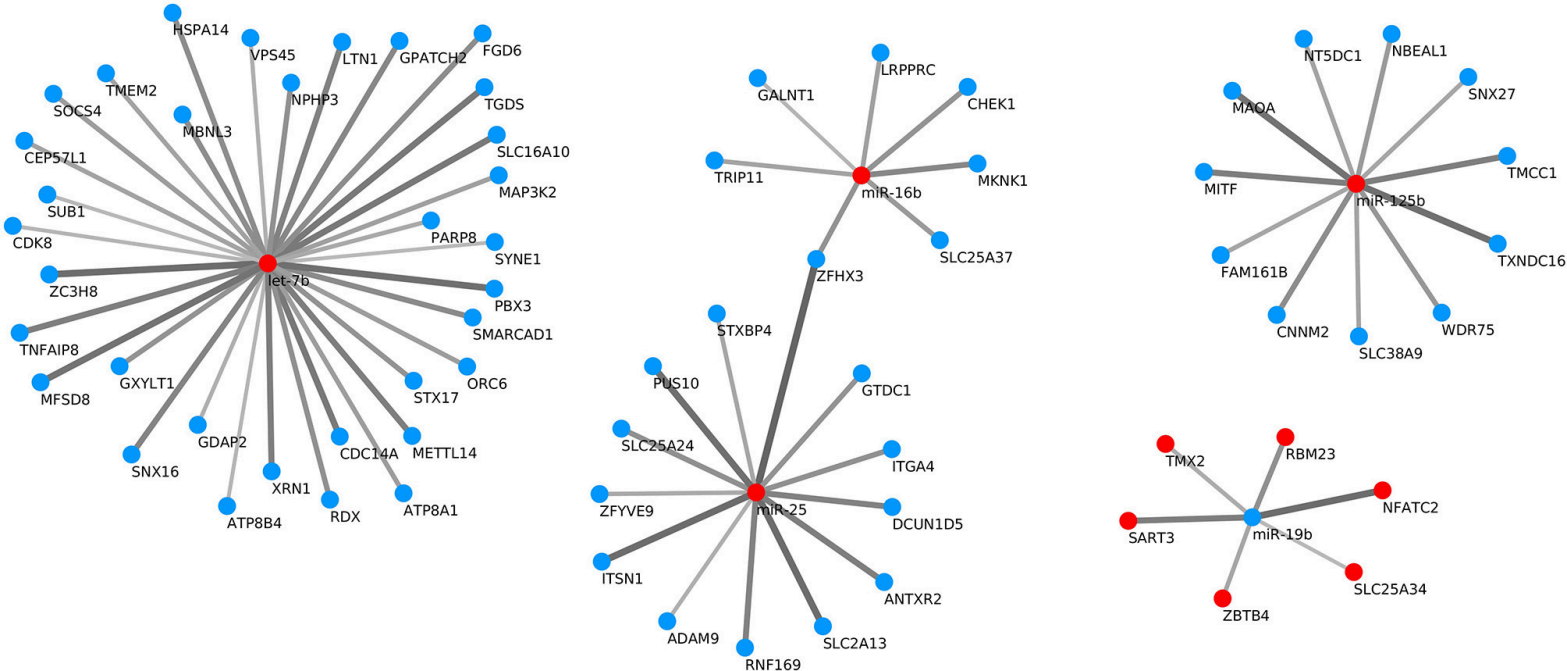
Significant negative correlations (with an adjusted *p*-value < 0.05) between differentially expressed miRNA-target pairs. Red points represent up-regulation and blue ones down-regulation of the miRNA or target gene. The greater the absolute value of the Spearman's rank correlation coefficient (rho), the broader and darker is the line joining the miRNA and predicted target.

Target gene predictions were performed for the differentially expressed miRNAs with three different programs (miRanda, PITA and TargetScan), taking the intersection of their results as potential targets. miRNAs usually act via translational repression and/or mRNA cleavage, although there is evidence of miRNAs upregulating translation by diverse mechanisms (37, 38). However, it must be determined whether the activation of protein translation is a general phenomenon or is only an exception in the mechanism of miRNA action. For that reason, when examining the miRNAs and their targets, only those miRNA-target pairs with negative correlation were selected for further study. Therefore, the miRNA expression data were integrated with the mRNA expression data to predict reliable miRNA-mRNA interactions, obtaining a total of 70 significant pairs with negative correlation (Figure 9). Among the miRNAs with more predicted targets, *oar-let-7b* had 33 predicted targets, followed by *oar-miR-25* and *oar-miR-125* with 13 and 11 predicted targets, respectively. The significant pairs (with a corrected *p*-value <0.05) had a Spearman's rank correlation coefficient (rho) value between −0.853 and −0.657.

## Discussion

Aluminum-based adjuvants, especially those containing aluminum hydroxide, are the most widely used adjuvants in human and animal vaccines (39, 40). Despite aluminum mineral salts being used as adjuvants for over 90 years, their mechanism of action is not totally understood. In the present study, RNA and miRNA sequencing were performed in PBMCs from lambs that were inoculated with commercial vaccines or with an equivalent quantity of aluminum hydroxide alone to study the molecular mechanisms of aluminum-based adjuvants. This is the first long-term *in vivo* study dealing with the molecular genetic basis of the immune response in sheep after repetitive inoculation with aluminum-containing vaccines or aluminum alone. Most studies on the immune response induced by aluminum have been done *in vitro*, analyzing the effects of adjuvants on immune system cells. This can help to define features of adjuvants that are essential for their function and to obtain a better understanding of mechanisms involved (13). However, it is crucial to understand the behavior of cells *in vivo* and the interactions they have with their environment to fully elucidate the mechanisms of action involved in aluminum adjuvant-induced responses. Aluminum adjuvants have a low dissolution and low elimination rate, especially the hydroxide-based adjuvant, and there have been different reports of low doses of aluminum remaining in the organism after long periods of time (41). Thus, there is a need for further long-term studies, as every year farm animals are inoculated with different aluminum-based vaccines, and it could be interesting to address the long-term effect that they might have (42).

This work presents some limitations inherent to the difficulties in the design of this kind of experiments. The number of animals analyzed by RNAseq is limited. In addition, animals that had been under treatment were analyzed only, and a longitudinal study was chosen in which the animals were analyzed at the beginning and end of the treatment, so that the final result of the repetitive experiment was appreciated with respect to the initial situation in each animal.

Moreover, priority has been given to the homogeneity of the individuals analyzed in the different groups, so young animals have been used, but all from the same herd, and without any vaccination before our experiment. Further, a period of adaptation to the new experimental flock was taken into account and they were in the best conditions of feeding and temperature, all of them controlled. Finally, as it is a repetitive vaccination experiment, it is very difficult to dissect the effect of each vaccine separately. We expect to see the cumulative effect of all the inoculations, without ruling out that the latter has a greater effect on the response of the animals than the previous ones.

The up- or downregulation of a number of genes that were previously described in other studies related to gene expression alteration in aluminum-induced response were also detected in this study, namely: *NLRP3, IL1B, IL8, TNF, NFKB2, RELA*, and *RELB*. *NLRP3* is a member of NLR family and is part of the molecular platform called inflammasome (43). There is a controversy about the requirements of *NLRP3* inflammasome in the aluminum-induced response (13, 44, 45). *In vitro*, aluminum-containing adjuvants stimulate the production of *IL1B*, and its production is dependent on the *NLRP3* inflammasome (46, 47). Despite the agreement on the involvement of *NLRP3* activation *in vitro*, how this translates to *in vivo* responses is controversial, and some studies have found no involvement of *NLRP3* in dendritic cell and lymphocyte activation by aluminum adjuvants (44, 47, 48). There is conflicting data about the necessity of the inflammasome to induce a humoral response (44, 46, 49), which is supposed to be predominant in aluminum based vaccines (50, 51). In our study, *NLRP3* was significantly downregulated in Adj-injected sheep. Thus, inflammasome does not seem to be necessary to induce an immune response in this *in vivo* experiment.

In addition, proinflammatory cytokines seem to have an important role in aluminum-induced response, especially when the antigen is present. Several reports have shown that secretion of inflammatory cytokines is induced by aluminum (47, 52–54). The consequent increase in inflammatory signals led to the activation of the *NF-*κ*B* signaling pathway. Interestingly, the expression levels of some *NF-*κ*B* family genes, such as *NFKB2, RELA* and *RELB*, were significantly increased in both the Vac Tf vs. Vac T0 and Adj Tf vs. Adj T0 comparisons. Lukiw et al. (55) also observed the upregulation of *NFKB2* in human neural cells exposed to aluminum. After activation, *NF-*κ*B* induces the transcription of proinflammatory mediators of the innate immune response, including the cytokines *TNF, IL1B*, and *IL8*. Kooijman et al. (56) also showed upregulation of *TNF* in human monocytes stimulated with aluminum hydroxide. Other studies found increased levels of *IL1B* mRNA expression in bovine PBMCs treated with aluminum (57). In our study, *IL1B* and *IL8* were significantly upregulated in Vac-injected sheep. Two other proinflammatory cytokines (*TNF* and *IL16*) have been found differentially upregulated in Adj-injected sheep, suggesting a nonspecific induction of proinflammatory responses when adjuvant alone is inoculated.

When we compared the most significant up- or downregulated genes, we observed that most of the genes related to apoptosis (*TP53BP2, CSRNP1, TEAD, CDCA7, PPP1R15A*) were upregulated in Vac- or Adj-injected sheep. This is in agreement with other studies, in which aluminum-induced apoptosis in the human neuroblastoma cell line (58) and the expression of pro-apoptotic genes in human brain cells (55) were also found. In addition, some genes related to the immune response (*SKAP2, IGSF6, LST1, FGR, MAPK13*), inflammatory response (*S100A12, ADGRE3, TREM1, STEAP4, NR4A3*), cell growth (*HGF, CSF3R*), and cell-cell signaling (*AREG*) were upregulated in Vac-injected sheep but were downregulated in Adj-injected animals. In contrast, some genes related to DNA replication and repair (*FEN1, HIST2H4A*) and involved in RNA binding, synthesis and metabolism (*IGF2BP3*) were downregulated in Vac-injected sheep and upregulated in Adj-injected sheep. In fact, aluminum stimulates the immune system by inducing immunological endogenous danger signals. Uric acid and host DNA have been shown to be released *in vivo* after aluminum injection (49, 59). Uric acid is released from the injured cells as a danger signal, which rapidly degrades RNA and DNA (49). Furthermore, among the most significant up- or downregulated genes, factors clearly related to nervous system development (*RAPGEF5, CASZ1, LICAM*) were upregulated in Adj-injected animals.

Interestingly, two autoimmune processes appeared in the pathway analysis, but in different comparisons: rheumatoid arthritis in the vaccinated sheep and systemic lupus erythematosus in the adjuvant-injected sheep and adjuvant vs. vaccine comparison. Therefore, it is possible that a previously described autoimmune syndrome in sheep (ASIA) (6) resembles these human autoimmune diseases and that the autoimmune effect of the adjuvant alone differs slightly from that obtained in combination with the antigens in vaccines.

Among the differentially expressed miRNAs, there were some previously described in other studies related to expression changes induced by aluminum, namely: *miR-19b* and *miR-125b*. *miR-125b* was upregulated after inoculation with commercial vaccines. *miR-125b* is a reactive oxygen species (ROS) and also a *NF-*κ*B* upregulated miRNA highly sensitive to aluminum-sulfate induction in stressed brain cells (60). Some of the aluminum-induced genes and miRNAs in brain cells exhibit expression patterns similar to those observed in Alzheimer's disease, with *miR-125b* being one of them (61, 62). In contrast, *miR-19b* was downregulated after inoculation with the adjuvant alone. Dysregulation of *miR-19b* is implicated in nervous system diseases, including Parkinson's disease (63, 64), and *miR-19b* is notably downregulated in the PBMCs of patients with Alzheimer's disease (65). miRNA pattern analysis links central nervous system damage pathways with the intensive vaccination protocol employed in this study, providing new insights on adverse effects after repetitive vaccination.

Within the negatively correlated targets of the differentially expressed miRNAs there are factors that are clearly related to the response to stimulus (*NBEAL1, CHEK1, MKNK1, ANTXR2, MAP3K2, HSPA14*), RNA binding (*WDR75, SART3, LRPPRC, SYNE1, RDX, XRN1, ZC3H8, SUB1, MBNL3*) and cellular response to DNA damage (*NFATC2, ZBTB4, STXBP4, RNF169*). In fact, aluminum-containing adjuvants induce endogenous danger signals (66) that can modulate immunity via cytotoxic effects (67). These endogenous danger signals, otherwise known as damage-associated molecular patterns (DAMPs), are released by necrotic cells and can subsequently induce alarm and inflammation (68) through recognition by pattern recognition receptors (PRRs). Aluminum produces granulomatous inflammatory reactions and promotes local necrosis in vaccinated muscle tissue (69) and in the peritoneum of mice following injection (59). Furthermore, a role for endogenous danger signals released during aluminum-induced cell death in driving immune responses has been demonstrated. In particular, host DNA has been shown to be released following aluminum injection (49). Marichal et al. (59) report that, in mice, aluminum causes cell death and the subsequent release of host cell DNA, which acts as a DAMP that mediates aluminum adjuvant activity.

In addition, some of the targets have been previously linked to the immune system. One of them is the *MAP3K2* (*MEKK2*) kinase gene, which is one of the predicted targets of the upregulated *let-7b* miRNA in Adj Tf vs. Adj T0. *MAP3K2* controls a delay in activation of *NF-*κ*B* in response to stimulation with proinflammatory cytokines and the formation of the *I*κ*B-*β*:NF-*κ*B:IKK* complex (70, 71). *SNX27* is another predicted target, in this case of the upregulated *miR-125b* in Vac Tf vs. Vac T0, whose silencing in human Jurkat T cells results in *NF-*κ*B* pathway hyperactivation (72). In the Vac Tf vs. Vac T0 comparison, *SNX27* is downregulated, and some *NF-*κ*B*-induced genes are upregulated. *CHEK1* is a predicted target of the upregulated *miR-16b* in Adj Tf vs. Adj T0. This gene is involved in DNA damage response. In accordance with our study, Farasani et al. (73) also found reduced levels of *CHEK1* in MCF10A-immortalized non-transformed human breast epithelial cells exposed to aluminum chloride or aluminum chlorohydrate, suggesting that aluminum can not only damage DNA but also compromise DNA repair systems.

In summary, this study demonstrated for the first time in a sheep model that aluminum adjuvants significantly increased the expression of inflammatory cytokines, *NF-*κ*B* family genes and apoptotic genes. The activation of the *NF-*κ*B* pathway might be regulated by miRNAs, such as *miR-125b* and *let-7b*. In addition, aluminum adjuvants play an important role in the *cytokine-cytokine receptor interaction pathway*. It was also revealed that aluminum affects genes related to DNA repair and cellular response to DNA damage stimulus. Due to the *NLRP3* gene downregulation in the Adj Tf group with respect to the Adj T0 group, inflammasome does not seem to be necessary for aluminum vaccines to induce an immune response, whereas DNA damage and uric acid are involved in the process.

Additionally, the overrepresentation of genes related to DNA damage stimulus and DNA repair in both groups may be due to miRNA-mediated regulation, as some of the predicted targets of the differentially expressed miRNAs are related to cellular response to DNA damage (e.g., *miR-16b* and its predicted target *CHEK1*). Furthermore, miRNAs, such as miR-25, miR-16b, and let-7b were associated with the aluminum adjuvant for the first time.

Taken together, these experiments demonstrate that aluminum containing adjuvants are not simply delivery vehicles for antigens but can also induce endogenous danger signals that can stimulate and modulate the immune system. Understanding vaccine factors that influence immune response has vast translational implications and may ultimately lead to the directed and rational development of new and more efficacious vaccine adjuvants with better immunogenicity and safety profiles.

## Data accession

The data discussed in this publication have been deposited in NCBI's Gene Expression Omnibus (GEO) and are accessible through GEO Series accession number GSE113899 (https://www.ncbi.nlm.nih.gov/geo/query/acc.cgi?acc=GSE113899).

## Author contributions

LL, DdA, and BJ conceptualization. BJ transcriptomics design, funding acquisition for transcriptomics and project administration. LL, JA, and MP animal management. JA, RR, DdA, and MP sample acquisition. NA and MS-P experimental analysis. EV-M bioinformatic analysis. NA experimental validation. EV-M, NA, and BJ visualization and analysis, writing–original draft. All authors writing–review and editing.

### Conflict of interest statement

The authors declare that the research was conducted in the absence of any commercial or financial relationships that could be construed as a potential conflict of interest.
