# Supplementary figures and images for: Molecular Signature of Aluminum Hydroxide Adjuvant in Ovine PBMCs by Integrated mRNA and microRNA Transcriptome Sequencing
Source: Front Immunol. 2018 Oct 23;9:2406. doi: 10.3389/fimmu.2018.02406 (PMC6206264; doi:10.3389/fimmu.2018.02406)

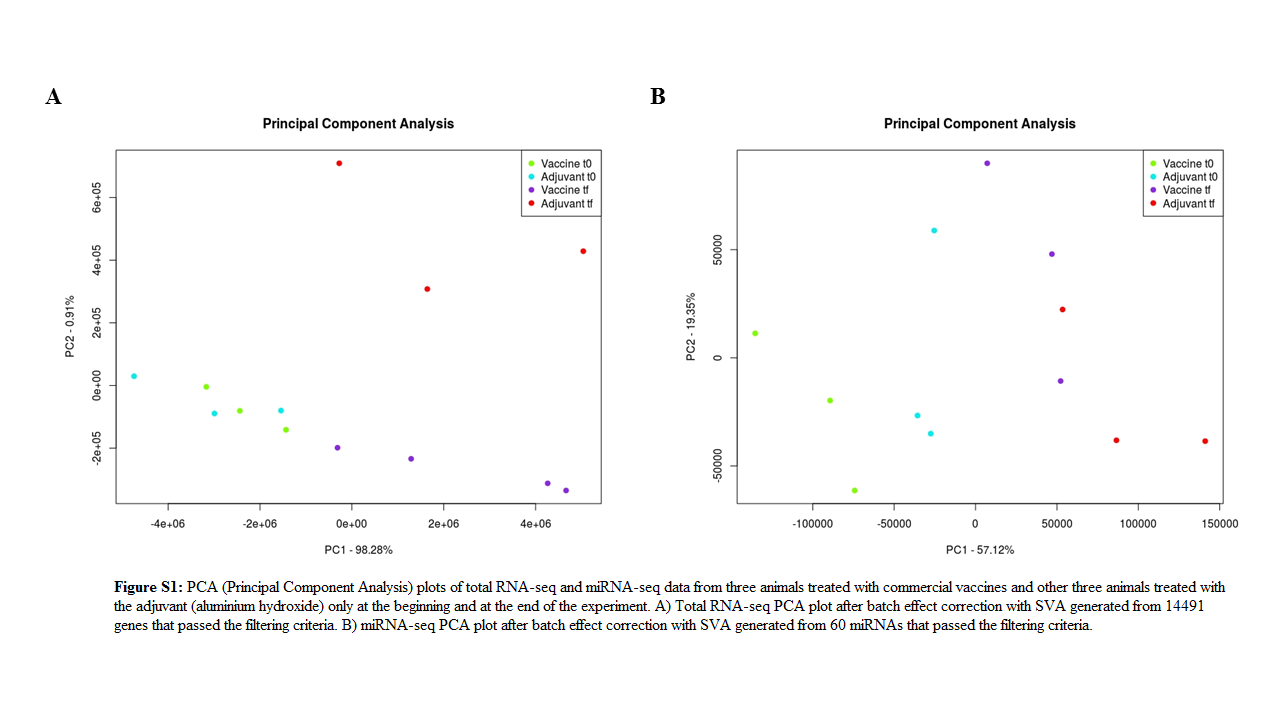

Supplement: Supplementary file 6 [file Image_1.tif]

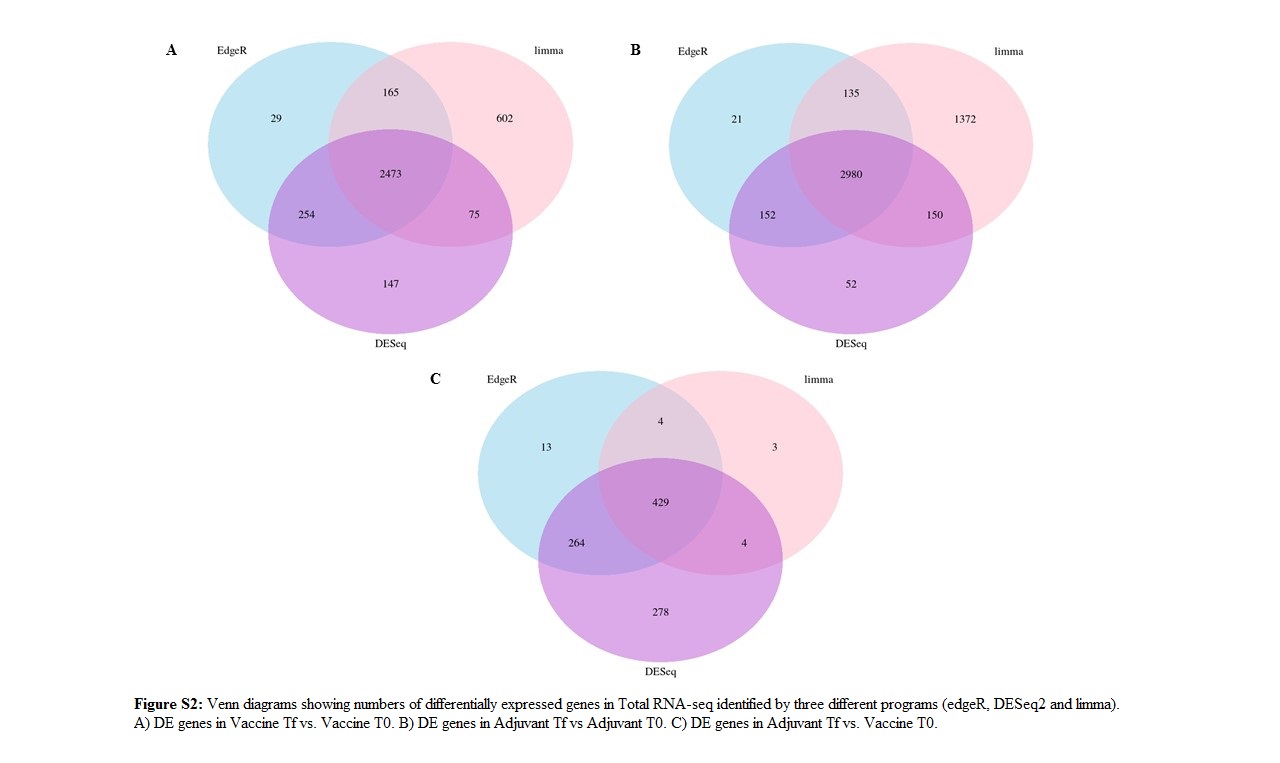

Supplement: Supplementary file 7 [file Image_2.jpg]
